# Parallel Analysis of Exosomes and Cytokines in Aqueous Humor Samples to Evaluate Biomarkers for Glaucoma

**DOI:** 10.3390/cells13121030

**Published:** 2024-06-13

**Authors:** Da Young Shin, Jeong-Sun Han, Chan Kee Park, Na Young Lee, Kyoung In Jung

**Affiliations:** 1 Department of Ophthalmology, College of Medicine, The Catholic University of Korea, Seoul 06591, Republic of Korea; eye-sdy1107@hanmail.net (D.Y.S.); winehan@catholic.ac.kr (J.-S.H.); ckpark@catholic.ac.kr (C.K.P.); 2 Eunpyeong St. Mary’s Hospital, Seoul 03312, Republic of Korea; 3 Seoul St. Mary’s Hospital, Seoul 06591, Republic of Korea

**Keywords:** glaucoma, biomarker, exosomes, cytokines

## Abstract

Recent emerging studies have demonstrated numerous critical roles of exosomes in cell-to-cell signaling. We investigated exosomes in the aqueous humor of glaucoma patients and controls and compared their characteristics with other biomarkers such as cytokines. Glaucoma patients exhibited higher exosome particle counts and smaller sizes compared to controls. Higher exosome density was correlated with more severe visual field loss. Conversely, concentrations of aqueous humor cytokines, particularly PD-L1, were primarily associated with intraocular pressure, and none of the cytokines showed a significant association with visual field damage. This may reflect the characteristics of exosomes, which are advantageous for crossing various biological barriers. Exosomes may contain more information about glaucoma functional damage occurring in the retina or optic nerve head. This highlights the potential importance of exosomes as signaling mediators distinct from other existing molecules.

## 1. Introduction

Glaucoma is a leading cause of vision loss, affecting >60 million persons globally in 2013, and is predicted to increase to 112 million by 2040 [1]. Glaucoma is characterized by the selective loss of retinal ganglion cells (RGCs). Increased intraocular pressure (IOP) is one of the major causative factors of glaucoma. However, glaucoma pathogenesis remains unclear in some respects because glaucoma can also develop within a normal IOP range and progress despite IOP-lowering treatment. Several mechanisms, including growth factor deprivation, oxidative stress, vascular dysregulation, excitotoxicity, and mitochondrial dysfunction, have been suggested as glaucoma pathophysiologies [2,3]. Identifying biomarkers for glaucoma pathogenesis could provide insights into its underlying mechanisms and might lead to the development of strategies for reducing its rate of progression through treatments associated with these biomarkers, in addition to reducing IOP.

Determining biomarkers in glaucoma patients through sampling the retinal tissue where RGCs are located or from the vitreous is a challenging task because vitrectomy is needed for tissue sampling in the posterior eye segment. Aqueous humor is more easily accessed for sampling by paracentesis through the cornea. It contains a range of growth factors and proinflammatory cytokines [4,5,6]. Cytokines are normally categorized as soluble factors that are of <30 kDa and include chemokines, interferons, interleukins, lymphokines, and the tumor necrosis factor (TNF) family of proteins, although cytokines may also function as a membrane-bound form [7,8]. Several cytokines and proteins, including transforming growth factor (TGF)-β, TNF-α, and monocyte chemotactic protein (MCP)-1, were found to be elevated in glaucoma patients [9,10,11,12,13].

Increased cytokines in the anterior chamber might originate in the anterior chamber itself or possibly from the posterior eye segment because it has been suggested that the cytokine level in the aqueous humor reflects that in the vitreous fluid to some degree [14]. In the anterior chamber, trabecular meshwork dysfunction and subsequent breakdown of the blood–aqueous barrier could be directly associated with the aqueous humor composition [15,16,17]. Regarding the posterior eye segment, neurodegeneration of RGC occurs in the optic nerve head and retina. Cytokines associated with glaucomatous pathophysiology could be secreted from glial cells or dysfunctional RGCs in terms of oxidative stress, neuroinflammation, or apoptosis [18]. In patients with primary open-angle glaucoma (POAG), many studies found that increased cytokines such as TGF-β2, TNF-α, MCP-1, or interleukin (IL)-8 were associated with higher IOP [9,13,19]. The relevance of cytokines in the aqueous humor to the degree of glaucomatous damage based on visual field (VF) tests is controversial [9,13,20]. Substantial amounts of soluble cytokines produced from glial cells or dysfunctional RGCs do not appear to travel to the aqueous humor without degradation and might thus not affect the aqueous humor composition.

The exosome is a type of extracellular vesicle that is nanosized (30–150 nm) with a double-layered phospholipid membrane and is found in various body fluids, including blood, urine, tears, vitreous humor, and aqueous humor [21,22]. Exosomes contain biomolecules, including proteins, mRNA, microRNA, transcription factors, and lipids [23]. Their double-layered membrane protects their cargo from enzymatic degradation, particularly in the body fluids, and also helps to regulate the interactions between exosomes and recipient cells [24]. Exosomes are able to cross biological barriers, including the blood–retinal barrier (BRB), because of their small size and membrane composition [21,25]. They are released by most cell types and facilitate essential biological functions such as cell-to-cell communication or biomolecular transport, locally and systemically [21,22].

Given these findings, it is possible that biomolecules in exosomes with their double membrane might endure longer than soluble cytokines when traveling from the retina or optic nerve head to the anterior chamber. Recently, exosome analysis found that patients with pseudoexfoliative glaucoma had a greater density of exosome particles than the control subjects [26]. Glaucomatous aqueous humor exosomes in patients with POAG were found to have a different phospholipid composition compared with the control exosomes [27]. However, a correlation between exosome characteristics and the degree of glaucomatous damage has, to our knowledge, not yet been demonstrated. In this study, we analyzed the exosome particles and their sizes in the aqueous humor of patients with POAG and the relationship between their characteristics and the degree of glaucomatous damage. We also analyzed cytokines in the aqueous humor to compare the significance of exosomes to that of such cytokines as a biomarker in glaucoma.

## 2. Materials and Methods

This study was approved by the Institutional Review Board of the Catholic University of Korea, Seoul, Republic of Korea (XC21TIDI0127). This study was performed in the Seoul St. Mary’s Hospital and Eunpyeong St. Mary’s Hospital of the Catholic University of Korea. This study followed the tenets of the Declaration of Helsinki and informed written consent was obtained from all study participants prior to their inclusion.

### 2.1. Subjects

This study enrolled consecutive patients with POAG who underwent glaucoma surgery or combined glaucoma surgery with cataract operation. Age-matched patients with cataracts but without glaucoma who underwent elective phacoemulsification were enrolled as the control subjects. The study inclusion criteria were the presence of an open-angle under gonioscopic examination, and an axial length < 30 mm. Exclusion criteria were corneal disease affecting vision; a history or current, uveitis, an optic neuropathy other than glaucoma, diabetic retinopathy, or other vitreoretinal diseases (e.g., retinal vein obstruction, central serous chorioretinopathy, age-related macular degeneration, retinal detachment); or brain disease that could impact visual function. Patients with no light perception or who were pregnant or breastfeeding were excluded. Further exclusion criteria included patients who underwent other combined ocular surgeries such as vitrectomy or penetrating keratoplasty.

### 2.2. Measurements

Enrolled patients preoperatively underwent a review of their medical history including central corneal thickness measurements by ultrasonic pachymetry, axial length measurements via ocular biometry, dilated fundus examinations, and Humphrey VF examinations using the Swedish interactive threshold standard 24-2 algorithm (Carl Zeiss Meditec, Inc., Dublin, CA, USA). Preoperative mean deviation (MD) and pattern standard deviation (SD) were analyzed using VF examinations performed within 1 month prior to surgery.

Peripapillary RNFL (retinal nerve fiber layer defect) thickness was measured with the Cirrus SD-OCT version 6.0 (Carl Zeiss Meditec, Inc., Dublin, CA, USA), using the Optic Disc Cube 200 × 200 scan mode. The protocol by which the peripapillary RNFL thickness was assessed was previously described in detail. Data of the mean RNFL thickness measurements were adopted in this study.

Long-term IOP fluctuation was determined as the SD of all IOP values measured during visits within 2 years preceding the surgeries. The peak IOP was defined as the highest IOP measured during visits within 2 years preceding the surgeries. The current IOP was the most recent IOP measurement taken prior to surgery.

A blood test, including a complete blood count, was performed routinely before surgery. We used variables such as the neutrophil-to-lymphocyte and platelet-to-lymphocyte ratios, which have been shown to be predictors for the prognosis of a systemic inflammatory condition [28].

Blood pressure (BP) was determined three times using an automated BP measurement device at the daycare center on the day of surgery; before surgery, immediately after surgery, and one hour after surgery. The brachial BP was measured in the supine position after the subject had been lying down for 5 min, with the arm in the appropriate position. The mean and SD of the systolic and diastolic BP were calculated from three measurements.

### 2.3. Sampling Procedure

At the time of surgery, approximately 50–150 µL of aqueous humor was collected from the anterior chamber under sterile conditions from the enrolled patients. Anterior chamber paracentesis was performed using a 30-guage needle in undertaking glaucoma surgeries glaucoma surgery phacoemulsification, or combined surgeries to obtain aqueous humor. Samples of aqueous humor were stored immediately at 4 °C and at −70 °C within three days until analysis. Due to the limited volume of aqueous humor available from each patient, it was necessary to perform the analyses for cytokines and exosomes separately on different patient groups.

### 2.4. Cytokine Analysis

Multiple cytokines were analyzed in the aqueous humor using a bead-based multiplex cytokine assay (R&D Luminex systems, Minneapolis, MN 55413, USA). Vascular endothelial-derived growth factor, MCP-1, GM-CSF, CXCL1, interferon-α, intercellular adhesion molecule-1, IL-1ra, IL-6, IL-7, IL-8, IL-15, CXCL10, CCL3, CCL4, PDGFAA, PD-L1, and TNF-β were determined using a Human XL Cytokine Luminex Performance Assay 46-plex Fixed Panel kit (R&D systems, Minneapolis, MN 55413, USA). TGF-β1 and -β2 levels were analyzed using a TGF-β Luminex Performance Assay 3-plex Kit. Data were collected and analyzed using a Bio-Plex 200 (Bio-Rad, Hercules, CA, USA) instrument equipped with Bio-Plex Manager analysis software (v. 6.1).

### 2.5. Exosome Isolation and ExoView Analysis

The aqueous humor samples were centrifuged at 3000× *g* and 4 °C for 30 min to remove the debris. Exosome Isolation Reagent (Exosome Plus, Seoul, Republic of Korea) was added to the AH supernatant. The isolation of exosomes was achieved using an Aqueous Two-Phase System (ATPS), composed of two polymers, dextran (DEX) and polyethylene glycol (PEG), supplemented with salts [29]. This system facilitates the separation of exosomes using mechanisms that involve hydrophobic and hydrophilic interactions, as well as van der Waals forces. These molecular interactions are critical for isolating exosomes with high purity and for preserving their structural integrity throughout the process. The mixtures were centrifugated at 3000× *g* for 10 min at 4 °C, resulting in two distinct phases separated by an interface: the top phase containing a PEG-rich solution and the bottom phase containing a DEX-rich solution. The PEG-rich phase was carefully pipetted into a new tube, while the solution near the phase interphase was separately removed. The remaining DEX-rich phase was also collected for further analysis. The pellet was resuspended in filtered phosphate-buffered saline and the diluted AH samples were rapidly placed on the chips (NanoView Bioscience, Boston, MA, USA), leaving them overnight at RT. Each chip was pre-coated with specific antibodies (CD9, CD63, CD81, and M IgG), and the analysis was performed with the Exoview human tetraspanin cargo kit (Nanoview Bioscience, Boston, MA, USA), which contained CD9, CD63, CD81, and syntenin antibodies, according to the manufacturer’s protocol [30]. The chips containing exosomes were washed with filtered water, dried carefully, and imaged on the ExoView™ R100 reader using ExoView Scanner version 3.2 acquisition software. The Exoview platform is designed to provide a detailed quantitative analysis of exosomes, displaying their size and concentration based on their surface protein markers. Sizing thresholds were set from a diameter of 50–200 nm. The data were analyzed with the ExoView Analyzer version 3.2. The volume of exosomes was calculated as “exosome count × exosome diameter^3^/100,000”.

### 2.6. Statistical Analysis

The statistical analysis for cytokines and exosomes was performed separately due to the limited amount of aqueous humor available from patients, which prevented simultaneous assessment of both. Statistical analyses were performed using the Statistical Package for the Social Sciences version 24.0 software program. Differences in parameters among the two groups were determined using Student’s *t* or chi-squared tests. Correlations between variables were calculated with Pearson correlation coefficients. Correlations of <0.4 were classified as weak, correlations of ≥0.4 and <0.6 as moderate, and correlations of ≥0.6 as strong [31]. A univariate and multivariate regression analysis was adopted to determine the factors associated with IOP or the MD of the VF. PCA analysis was used to summarize various individual factors into simple groups with similar properties. We used direct oblimin rotation to allow for correlations between components. The PCA results were visualized using biplots that display both the loadings of variables on the principal components and the scores of observations on these components. *p*-values of <0.05 were chosen to indicate statistically significant differences.

## 3. Results

### 3.1. Exosomes in Aqueous Humor

A total of 34 eyes of 34 patients were included in the exosome analysis. Eighteen eyes were diagnosed with POAG, and 16 control eyes had cataracts without glaucoma. The demographics of the included subjects are presented in Table 1. The mean age of the subjects with POAG and control subjects was 62.9 ± 13.3 years and 63.6 ± 10.6 years, respectively (*p* = 0.883). The baseline MD was −16.16 ± 10.67 dB in the POAG group and −1.90 ± 1.88 dB in the control group (*p* < 0.001). The POAG group had higher IOP than the control group with respect to the current IOP (25.91 ± 9.25 mmHg vs. 15.74 ± 1.98 mmHg, *p* < 0.001), peak IOP (35.39 ± 1.65 mmHg vs. 15.67 ± 1.92.1 mmHg, *p* < 0.001), and SD IOP (5.34 ± 5.72 mmHg vs. 1.27 ± 0.99 mmHg, *p* < 0.001). In the POAG group, 12 patients had phakic eyes (66.7%) and 6 patients had pseudophakic eyes (33.3%). In the control group, all patients had phakic eyes, because they exhibited no ocular comorbidities other than simple cataracts.

**Figure 1:**
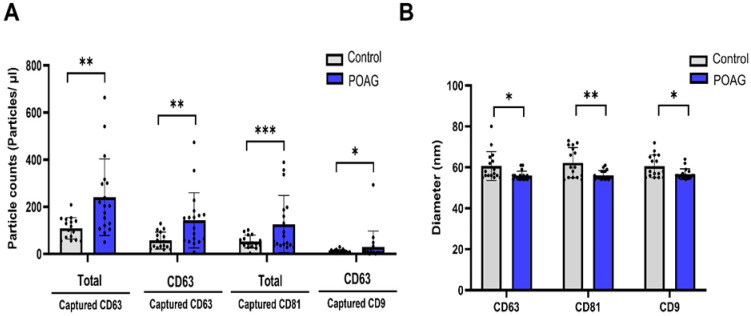
The number and diameter of exosome particles in grouped clinical data. (**A**) Compared to the control subjects (black colored in the bar graph), the numbers of the exosome particles in primary open-angle glaucoma (POAG) patient aqueous humor (AH) samples (blue colored in the bar graph) were higher, with statistically significant differences. (**B**) The diameter of exosome particles in grouped clinical data. Compared to the control subjects (black colored in the bar graph), the diameters of the exosome particles in POAG patient AH samples (blue colored in the bar graph) were smaller, with statistically significant differences. * *p* < 0.05, ** *p* < 0.01, *** *p* < 0.001.

Exosome particle counts tended to be higher in the POAG than in the control group (Table 2). Captured CD63 (total), CD 63 (CD63), captured CD 81 (total), and captured CD9 (CD63) concentrations were higher in the POAG group than the control group (*p* = 0.004, 0.008, <0.001, and 0.037, respectively, Figure 1A). The mean exosome diameters for CD63, CD81, and CD9 were all smaller in POAG patients compared with controls (Table 2, Figure 1B). The volume of exosomes tended to be larger in the POAG group than in the control group, but this was only statistically significantly different in CD63 exosomes (*p* = 0.039).

In the regression analysis to determine the factors associated with glaucoma severity, the higher exosome particle count measured based on captured CD63 (CD63) was associated with a lower MD of the VF (*p* = 0.016, Table 3).

**Figure 2:**
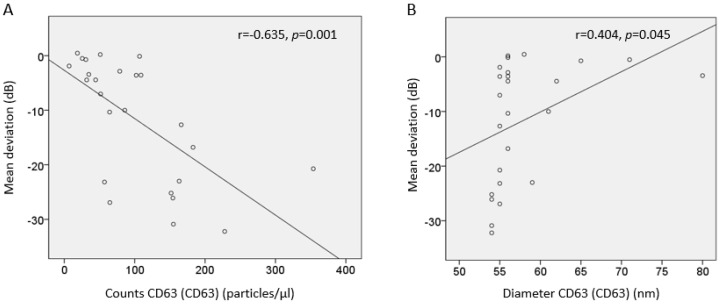
Scatter plot of the correlation between aqueous exosome particle count and size measured by CD63 (CD63) marker and mean deviation of visual field tests. (**A**) The number of exosomes was negatively correlated with mean deviation (r = −0.635, *p* = 0.001). (**B**) The smaller the size of exosomes, the lower the mean deviation (r = 0.404, *p* = 0.045).

The correlation between exosome counts and the degree of VF damage was found to be moderate (r = 0.635, *p* = 0.001; Figure 2). The correlation between exosome size and the degree of VF damage was found to be nearly weak (r = 0.404, *p* = 0.045).

In the regression analysis to investigate the factors associated with the exosome count (captured CD63), the MD of the VF remained as the associated factor (*p* = 0.014, Table 4).

Regression analysis was performed to determine IOP and associated factors, but no relationship with exosomes was found. Only age displayed a relationship with the current IOP in multivariate linear regression analysis (*p* = 0.032, Supplemental Table S1). In the regression analysis with the SD of IOP, no statistical association was found (Supplemental Table S2).

**Figure 3:**
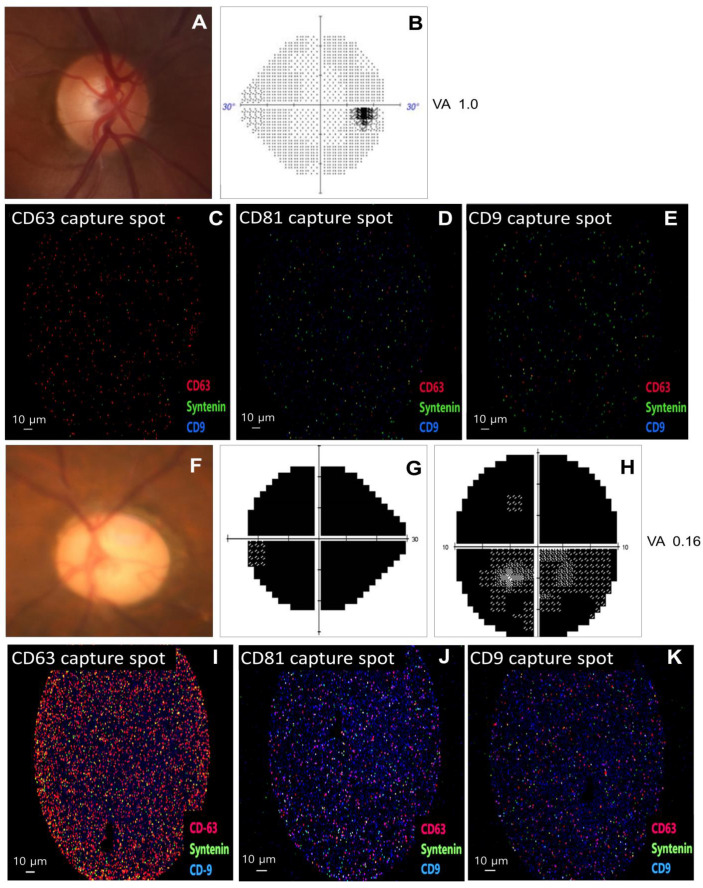
Representative images showing capture spots (CD63, CD81, and CD9) and clinical examinations in a control subject (**A**–**E**) and primary open-angle glaucoma (POAG) patient (**F**–**K**). The control subject showed a normal optic disc (**A**), a normal visual field (VF) test result (**B**), and capture spots showing exosomes labeled with CD 63 (red), syntenin (green), and CD9 (blue) (**C**–**E**). The POAG patient exhibited severe optic disc cupping (**F**) and advanced VF loss (**G**,**H**). Capture spots (**I**–**K**) reveal exosomes labeled with CD63, syntenin, and CD9, with an increased number of exosome particles compared to the control subject. Additionally, among each tetraspanin capture antibody detected in each capture spot, CD63 was present in the highest numbers of the exosomes, followed by syntenin and CD9.

Figure 3 displays representative cases from both a control subject and a POAG patient. In a control subject, capture spot images of the subject’s aqueous humor showed exosomes labeled with CD 63, syntenin, and CD 9, indicating the presence of these markers in the exosome population (Figure 3A–E). The POAG patients showed severe optic disc cupping and advanced VF damage. Capture spot images of the POAG patient’s aqueous humor demonstrated an increased number of exosome particles compared to the control subject. These exosomes were also labeled with CD63, syntenin, and CD9, with CD63 being the most abundant, followed by syntenin and CD9.

In POAG patients, the eigenvalue of the first principal component (PC1) was 9.359, accounting for 78.0% of the total variability in the data (Supplemental Figure S1). The eigenvalue of the second principal component (PC2) was 1.428 and PC2 accounted for 11.9% of all variability in the data. The cumulative contribution rate of PC1 and PC2 was 89.9%. All exosome concentrations had positive principal component loadings (PCLs) in PC1, and all these loadings were above 0.7. Captured CD63s (total, CD63, syntenin, CD9) showed remarkably elevated positive values in PC1 of POAG patients while captured CD63s (total, CD63) exhibited negative values in PC2 of control subjects.

### 3.2. Cytokines in Aqueous Humor

A total of 48 eyes of 48 patients were included in the cytokine analysis. Eighteen eyes were diagnosed with POAG, and thirty control eyes had cataracts without glaucoma. The demographics of the included subjects are presented in Table 5. There was no significant difference in the mean age between the two groups (*p* = 0.231). The POAG group had a higher proportion of men (72.2%), whereas the control group had a higher proportion of women (70%, *p* = 0.007). The baseline MD was −16.6 ± 9.7 dB in the POAG group and −4.9 ± 3.7 dB in the control group (*p* < 0.001). The POAG group compared with the control group had a higher current IOP (27.6 ± 8.7 mmHg vs. 15.4 ± 2.7 mmHg, *p* < 0.001), peak IOP (31.1 ± 9.3 mmHg vs. 16.27 ± 4.1 mmHg, *p* < 0.001), and SD of IOP (4.7 ± 2.7 mmHg vs. 1.4 ± 1.3 mmHg, *p* < 0.001). In the POAG group, 10 patients had phakic eyes (55.6%) and 8 patients had pseudophakic eyes (44.4%). In the control group, all patients had phakic eyes because they had no ocular comorbidities other than simple cataracts.

In the analysis of cytokines, the TGF-β1, TGF-β2, MCP-1, IL-7, IL-15, Platelet-Derived Growth Factor AA, and programmed death-ligand-1 (PD-L1) concentrations displayed statistical differences between the two groups (*p* = 0.003, 0.001, 0.005, <0.001, 0.001, 0.002, <0.001, respectively; Table 6).

In multivariate analysis to determine the factors associated with IOP, all systemic variables described in the demographics, and cytokines that displayed significant differences between the two groups were included. Lens status and the aqueous humor PD-L1 cytokine level were associated with the current IOP level on multivariate linear regression analysis (*p* < 0.001, *p* = 0.001, respectively, Table 7).

**Figure 4:**
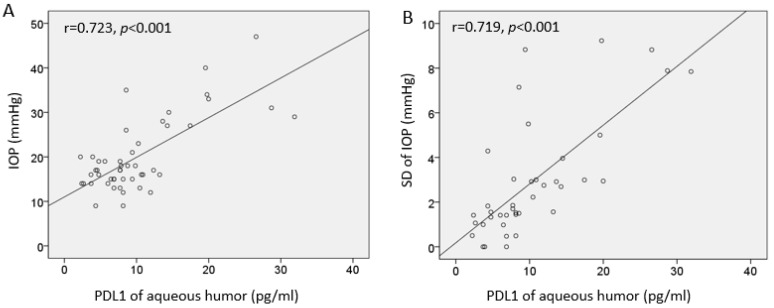
Relationship between aqueous PD-L1 level and intraocular pressure (IOP). (**A**) PD-L1 concentration was positively correlated with IOP (r = 0.723, *p* < 0.001). (**B**) Greater PD-L1 level was correlated with standard deviation of IOP (r = 0.719, *p* < 0.001).

The PD-L1 concentration was positively correlated with IOP (r = 0.723, *p* < 0.001, Figure 4). In the regression analysis, PD-L1 and SD of IOP displayed a strong correlation with each other (Supplemental Tables S3 and S4). No statistical association was found in the regression analysis with the MD of the VF (Supplemental Table S5).

In principal component analysis (PCA), the eigenvalue of the PC1 was 8.225, which accounts for 45.7% of the total variability in the data (Supplemental Figure S2). The eigenvalue of PC2 was 3.752, contributing 20.8% to the overall variability. For POAG patients, cytokines such as PD-L1, IL-6, IL-7, and MCP-1 showed higher loadings in PC1. PD-L1 showed negative values in PC1 and PC2 for control subjects.

## 4. Discussion

In this study, we observed higher levels of exosomes in aqueous humor of glaucoma patients compared to controls, and their increased concentration was associated with more severe glaucomatous VF damage. Cytokine analysis did not reveal a significant correlation with the degree of VF loss, while greater PD-L-1 concentration was associated with higher IOP.

The higher exosome particle counts observed in patients with POAG in our study suggest a novel finding, as there has been no previous study reporting the concentration of exosomes in POAG. These results are consistent with findings from An et al., who reported a greater number of exosomes in pseudoexfoliative glaucoma compared to the control group [26]. The exact mechanism underlying the exosome concentrations in the glaucoma group remains unclear, but several hypotheses warrant consideration. Firstly, the pathophysiology associated with elevated IOP in patients with glaucoma may contribute to the increased exosome particle counts. Elevated IOP arises from trabecular meshwork dysfunction, which could potentially trigger the release of exosomes in the anterior chamber [32]. However, it is noteworthy that the correlation between IOP and the concentration of exosomes was not statistically significant in the multivariate analysis. This suggests that factors beyond elevated IOP may influence exosome levels in glaucoma. Secondly, we identified a significant association between higher aqueous humor exosome particle counts and more severe VF loss in our study. As VF loss progresses, it typically corresponds to increased RGC loss. It is plausible that exosomes, secreted from glial cells or dysfunctional RGCs during the pathogenesis of glaucoma, might contribute to elevated exosome concentration observed in the anterior chamber [33]. The correlation between exosome counts and the degree of VF damage was found to be moderate (r = 0.635, *p* = 0.001), as depicted in Figure 2. While this moderate correlation suggests a certain level of predictability between these two variables, it also indicates that other factors likely influence their relationship. This observation underscores the potential of exosome counts as biomarkers for glaucoma pathogenesis, yet also highlights the need for further studies to explore the consistency of these findings and to validate exosome counts as reliable predictors of progressive VF loss.

We observed not only changes in the number of exosomes but also a shift in the distribution of EVs toward smaller configurations. Specifically, the size of exosomes was overall smaller in glaucoma patients compared to controls. Mueller et al. also demonstrated a reduction in the particle size of aqueous humor exosomes in POAG patients compared with control subjects, whereas another study found no difference in exosome size between pseudoexfoliation glaucoma patients and controls [26,27]. Studies in cancer have indicated that low oxygen concentration contributes to a decrease in the size of exosomes [34]. It is conceivable that factors inherent to glaucoma pathophysiologies, such as growth factor deprivation, oxidative stress, and vascular dysregulation might influence the exosome size. Smaller exosome particles may have the ability to traverse the blood–brain barrier more rapidly under pathological conditions and could be taken up more swiftly by target cells [34,35]. Additionally, it has been reported that exosomes with higher density exhibit smaller sizes compared to those found in the low-density fractions [36]. Therefore, the elevated exosome concentration observed in glaucoma patients might be related to smaller exosome sizes, potentially facilitating their transfer from the posterior segment to the anterior chamber. However, the correlation between exosome size and the degree of VF damage was found to be nearly weak (r = 0.404, *p* = 0.045) and the exosome size did not remain a significant factor associated with VF MD. The results imply that the smaller exosome size observed in POAG might be a secondary effect, possibly linked to higher exosome counts rather than a direct causative factor in VF damage. This indicates that while exosome size may contribute to our understanding of the disease process, it does not independently predict the severity of VF loss.

Exosomes play a crucial role in intercellular communication by transporting biomolecules such as proteins, mRNA, microRNA, and lipids. Understanding the cargo carried by exosomes is essential, but the limited volume of aqueous humor poses challenges for in-depth analysis of their contents. Therefore, we employed the volume index to estimate the total amount of exosomes using the diameter cubed as a proxy. Our findings revealed that the volume of exosomes tended to be larger in the POAG group compared to the control group, although the volume index did not remain as a factor associated with the degree of visual field loss in multivariate analysis. This indicated that not only the number but also the total amount of exosomes increased in glaucoma. Further research is needed to elucidate the precise mechanisms underlying these observations.

The study selected the exosome markers CD63, CD81, and CD9 due to their prominent role as tetraspanin proteins, which are specially enriched in the membrane of exosomes [37,38]. This makes them reliable markers for exosome detection and characterization. Exosomes can fuse with the plasma membrane to deliver their cargo to a recipient cell, facilitated by tetraspanin complexes, which include CD9, CD63, and CD 81. Additionally, An et al. demonstrated that exosomes derived from aqueous humor, detected by these tetraspanins, might play a role in the pathogenesis of pseudoexfoliative glaucoma [26]. This supports the use of these markers in identifying and characterizing exosomes in glaucoma research. This study employed ExoView™ R100 platform to analyze exosomes, using capture spots coated with specific antibodies targeting CD 63, CD81, and CD9. After exosomes are captured, immunofluorescent labeling with the Exoview human tetraspanin cargo kit (containing CD9, CD63, CD81, and syntenin antibodies) is used to detect and visualize these proteins, providing information on their co-expression and relative abundance on individual exosomes. Detecting multiple tetraspanins simultaneously enhances the specificity of exosome detection by reducing false positives and distinguishing between different exosome subpopulations. We found a significant association between exosome particle counts and the degree of VF damage when the capture antibody was detected on the CD 63 capture spot. Previous research observed in patients with pseudoexfoliative glaucoma also noted the highest number of exosomes detected using antibodies targeting CD63 among tetraspanin complexes [26]. Several studies have reported that exosomes can effectively cross the blood–brain barrier when the tetraspanin surface protein CD63 is abundant on the exosome. CD63 is a key protein utilized in engineered exosomes to improve cargo loading and enhance reporter efficiency [39]. These unique properties may contribute to exosomes moving more effectively from the retina or optic nerve head to the anterior chamber, offering insights into their functional roles in glaucoma pathogenesis.

In our study, we found a positive correlation between aqueous humor cytokines and IOP. IOP is influenced by the production and drainage of aqueous humor, primarily through the trabecular meshwork. Given that abnormalities in the trabecular meshwork are associated with changes in IOP, cytokines levels may reflect the anterior environment of the eye. The significant association between IOP and cytokine concentrations aligns with previous research indicating that higher inflammatory cytokine concentrations were linked to elevated IOP in patients with glaucoma [10,40,41]. We also investigated the potential impact of anti-glaucoma eyedrops on cytokine concentrations and found no significant relationship between the number of glaucoma eye drops and IOP.

Interestingly, among various cytokines examined, PD-L1 was elevated in glaucoma patients and showed a significant association with increased IOP or greater IOP fluctuation in our multivariate analysis. PD-1, as a negative regulator of the immune response, and its ligand PD-L1 expressed on both hematopoietic and non-hematopoietic cells, have been found to suppress the central nervous system immune response via microglia and infiltrating macrophages [42]. Our study provides novel insights by examining PD-L1 levels in the aqueous humor of glaucoma patients, a focus not previously explored in the literature. The observed increase in PD-L1 may suggest its involvement in neuroinflammatory processes within the ocular environment in glaucoma. Notably, Sheng et al. demonstrated that blocking both the PD-L1/PD-L2 pathways had a protective effect on RGCs and increased the number of anti-inflammatory M2-activated microglia in a mouse model of chronic ocular hypertension [43]. This suggests that the observed increase in PD-L1 in our study may be indicative of neuroinflammation in glaucoma patients, potentially offering a new avenue for understanding disease mechanisms or developing therapeutic targets [44]. PD-L1 has been implicated also in the induction of fibrosis in pulmonary hypertension [45], suggesting that increased PD-L1 levels may be indicative of trabecular meshwork fibrosis, though further studies are warranted to clarify this relationship. In our study, we did not find any cytokine in the aqueous humor associated with the severity of glaucoma. This may indicate that cytokines face challenges in moving to the anterior chamber without degradation in the posterior segment, unlike exosomes.

PCA was utilized to simplify the analysis of complex biomarkers, including exosomes and cytokines, to identify patterns and to compare the molecular profiles between POAG patients and control subjects [46]. This analysis revealed that the PC1, accounting for 78.0% of the variance, was strongly associated with elevated levels of exosome markers such as CD63, syntenin, and CD9 in POAG patients. This suggests a significant role for exosomes in the pathophysiology of POAG, particularly since captured CD 63 markers were negatively loaded in control subjects. Similarly, the cytokine analysis through PCA revealed distinct profiles between POAG patients and controls. PC1, which accounted for 45.7% of the variance, was highly influenced by cytokines such as PD-L1, IL-6, IL-7, and MCP-1. The marked elevation of these cytokines in POAG patients within PC1, coupled with the negative loadings of PD-L1 in controls, underscores the significance of PD-L1 in the pathophysiology of glaucoma.

Due to the limited volume of aqueous humor available from each patient, we conducted separate analyses for exosomes and cytokines. As hypothesized in the introduction, biomolecules within the double-membrane structure of exosomes may endure longer than soluble cytokines when traveling from the retina or optic nerve head to the anterior chamber. By analyzing these components in a complementary manner, we aimed to provide a more comprehensive overview of the molecular landscape in the aqueous humor of glaucoma patients, enhancing our understanding of their potential interplay and collective impact on the disease’s pathophysiology.

Our study has several limitations. Firstly, our results may not be applicable to all glaucoma patients because we only included individuals of Korean descent. Secondly, we solely investigated the number, size, and volume of exosomes whereas a comprehensive understanding of their role requires analysis of their components. Unfortunately, the limited volume of aqueous humor samples obtained poses challenges for simultaneously conducting comprehensive analyses of both exosome number and their contents. There are few studies on aqueous humor exosomes, with some combining samples from only a few patients [23,47,48]. Further research is necessary to identify the components of exosomes in aqueous humor. Lastly, although we observed a positive correlation between exosome counts and RGC loss as measured by MD, it remains unclear whether a higher density of exosomes contributes to glaucomatous damage or results from it. Further investigation is necessary both to determine the causal relationship between exosome characteristics and progressive VF loss in glaucoma and to clarify how these characteristics relate to disease progression, potentially validating their use as biomarkers for early detection and ongoing monitoring of glaucoma.

## 5. Conclusions

In conclusion, our study demonstrated a positive correlation between the number of exosomes and glaucoma severity, underscoring the potential significance of exosomes as signaling mediators distinct from other existing molecules. This suggests that the exosomes of the aqueous humor in the anterior chamber may provide insights into the posterior environment, particularly regarding key sites of glaucoma pathophysiology such as RGCs or the RNFL. Furthermore, our findings suggest that exosomes hold promise as a potential treatment modality for glaucoma. By serving as carriers of biological information, exosomes may offer targeted interventions that could modulate disease progression at a molecular level.

## Figures and Tables

**Table 1:** Demographics according to the presence of glaucoma in the subjects being analyzed for exosome characterization.

Characteristics		Control (n = 16)	POAG (n = 18)	*p* Value
Age (years)		63.6 ± 10.6	62.9 ± 13.3	0.883
Sex (male/female)		7/9	10/8	0.492
Diabetes, n (%)		2 (12.5%)	6 (33.3%)	0.153
Hypertension, n (%)		1 (6.3%)	5 (27.8%)	0.100
Heart Coronary disease, n (%)		1 (6.3%)	0 (0%)	0.282
Mean SBP (mmHg)		137.02 ± 12.48	137.69 ± 15.20	0.889
Mean DBP (mmHg)		76.10 ± 6.87	77.02 ± 7.74	0.720
SD of SBP (mmHg)		8.11 ± 6.50	7.50 ± 4.87	0.760
SD of DBP (mmHg)		4.79 ± 2.76	5.72 ± 5.04	0.513
NLR		2.29 ± 1.07	2.49 ± 1.86	0.707
PLR		130.77 ± 69.82	120.98 ± 52.16	0.644
CCT (µm)		543.30 ± 37.93	528.44 ± 25.55	0.208
Axial length (mm)		25.00 ± 1.95	25.72 ± 1.79	0.285
Current IOP (mmHg)		15.74 ± 1.98	25.91 ± 9.25	**<0.001**
Peak IOP (mmHg)		15.67 ± 1.92	35.39 ± 1.65	**<0.001**
SD of IOP (mmHg)		1.27 ± 0.99	5.34 ± 5.72	**<0.001**
Average RNFL thickness		87.75 ± 7.15	64.18 ± 14.75	**<0.001**
Number of eyedrops		0.3 ± 0.6	3.5 ± 0.9	**<0.001**
PG analogues		0.1 ± 0.3	0.9 ± 0.3	**<0.001**
Lens status	Phakic	16	12	**0.011**
	Pseudophakic	0	6	
Visual field 24-2	MD (dB)	−1.90 ± 1.88	−16.16 ± 10.67	**<0.001**
	PSD (dB)	2.29 ± 1.23	6.50 ± 3.30	**<0.001**

CCT, Central corneal thickness; DBP, diastolic blood pressure; IOP, intraocular pressure; MD, mean deviation; NLR, neutrophil-to-lymphocyte ratio; PG, prostaglandin; PLR, platelet-to-lymphocyte ratio; POAG, primary open-angle glaucoma; PSD, pattern standard deviation; RNFL, retinal nerve fiber layer; SBP, systolic blood pressure; SD, standard deviation. Student’s *t*-test for continuous variables and chi-squared test for categorical variables. Statistically significant differences between groups are indicated in bold.

**Table 2:** Exosome characterization in patients with and without glaucoma.

Exosome			Control (n = 16)	POAG (n = 18)	*p* Value
Particle counts (particles/μL)	Captured CD63	Total	108.98 ± 45.48	240.29 ± 162.82	**0.004**
		CD63	56.76 ± 35.86	142.41 ± 117.05	**0.008**
		Syntenin	9.82 ± 6.12	35.73 ± 56.78	0.079
		CD9	11.47 ± 7.04	28.73 ± 68.47	0.609
	Captured CD81	Total	52.06 ± 27.37	126.38 ± 121.03	**<0.001**
		CD63	9.49 ± 6.81	19.50 ± 26.44	0.151
		Syntenin	8.60 ± 8.59	16.00 ± 30.59	0.357
		CD9	20.11 ± 12.41	28.89 ± 37.46	0.378
	Captured CD9	Total	87.53 ± 64.16	127.80 ± 140.81	0.099
		CD63	11.47 ± 7.04	22.83 ± 42.52	**0.037**
		Syntenin	11.45 ± 9.32	21.56 ± 51.69	0.076
		CD9	38.78 ± 51.71	35.05 ± 81.05	0.876
Diameter (nm)		CD 63	60.60 ± 37.03	55.94 ± 2.18	**0.011**
		CD 81	62.25 ± 7.33	56.17 ± 2.23	**0.002**
		CD 9	60.56 ± 5.53	56.67 ± 2.61	**0.012**
Volume (particles·nm^3^)/100,000		CD 63	241.52 ± 113.67	434.44 ± 342.52	**0.039**
		CD 81	122.82 ± 69.59	228.87 ± 227.59	0.083
		CD 9	198.43 ± 174.52	256.91 ± 363.71	0.562

POAG, primary open-angle glaucoma. Statistically significant differences between groups are indicated in bold.

**Table 3:** Univariate and multivariate analysis of factors associated with mean deviation of visual field.

Parameter	Characteristics	Univariate	*p* Value	Multivariate	*p* Value
		β Coefficient (95% CI)		β Coefficient (95% CI)	
Systemic factors	Age	0.021 (−0.373~0.415)	0.913		
	Gender	0.283 (−9.161~9.726)	0.951		
	Diabetes	−11.087 (−21.757~−0.387)	**0.043**	−3.983 (−13.845~5.879)	0.405
	Hypertension	−9.276 (−19.498~0.945)	0.073		
	Heart coronary disease	8.509 (15.131~32.149)	0.464		
	Mean SBP (mmHg)	−0.040 (−0.368~0.289)	0.806		
	Mean DBP (mmHg)	0.041 (−0.625~0.708)	0.899		
	SD of SBP (mmHg)	0.335 (−0.699~1.368)	0.510		
	SD of DBP (mmHg)	−0.328 (−1.406~0.749)	0.535		
	NLR	0.564 (−2.416~3.545)	0.699		
	PLR	0.026 (−0.069~0.121)	0.575		
	Current IOP	−0.331 (−0.658~−0.004)	**0.048**	1.011 (−13.845~5.879)	0.224
	Peak IOP	−0.358 (−0.684~−0.033)	**0.032**	−0.982 (−2.718~0.754)	0.248
Ocular factors	Axial length	−0.689 (−3.476~2.099)	0.613		
	CCT	0.013 (−0.164~0.190)	0.880		
	Lens status	−11.592 (−21.364~−1.820)	**0.022**	−2.262 (−12.156~7.633)	0.635
	Number of eyedrops	−3.947 (−6.272~−1.621)	**0.002**	−2.378 (−7.654~2.898)	0.354
	PG analogues	−10.606 (−19.554~−1.658)	**0.022**	−2.016 (−17.352~13.320)	0.784
Exosome particle counts	CD 63 (total)	−0.037 (−0.074~0.000)	**0.049**		
	CD 63 (CD63)	−0.088 (−0.135~−0.042)	**0.001**	−0.071 (−0.126~−0.015)	**0.016**
	CD 81 (total)	−0.006 (−0.058~0.047)	**0.003**		
Exosome diameter	CD 63	0.738 (0.017~1.460)	**0.045**	−0.067 (−0.126~−0.015)	0.859
	CD 81	0.725 (−0.025~1.475)	0.057		
	CD 9	0.895 (−0.103~1.893)	0.076		
Exosome volume	CD 63	−0.014 (−0.037~0.009)	0.226		
	CD 81	0.003 (−0.025~0.030)	0.840		
	CD 9	0.006 (−0.024~0.036)	0.690		

CCT, central corneal thickness; DBP, diastolic blood pressure; IOP, intraocular pressure; NLR, neutrophil-to-lymphocyte ratio; PLR, platelet-to-lymphocyte ratio; SBP, systolic blood pressure; SD, standard deviation. The statistically significant variables in the regression analysis were indicated in bold.

**Table 4:** Multivariate analysis of factors associated with exosome particle counts (CD63 CD63).

Parameter	Characteristics	Univariate	*p* Value	Multivariate	*p* Value
		β Coefficient (95% CI)		β Coefficient (95% CI)	
Systemic factors	Age	0.140 (−2.798~3.078)	0.923		
	Gender	−1.594 (−70.836~67.647)	0.963		
	Diabetes	75.788 (−1.136~152.711)	0.053		
	Hypertension	29.616 (−60.575~119.806)	0.508		
	Heart coronary disease	−69.383 (−272.769~134.003)	0.492		
	Mean SBP (mmHg)	−0.794 (−3.328~1.740)	0.528		
	Mean DBP (mmHg)	−0.637 (−5.479~4.205)	0.790		
	SD of SBP (mmHg)	−0.381 (−6.643~5.881)	0.902		
	SD of DBP (mmHg)	−4.180 (−12.626~4.267)	0.321		
	NLR	16.635 (−5.714~38.984)	0.139		
	PLR	0.228 (−0.348~0.805)	0.426		
Ocular factors	Current IOP	3.038 (0.530~5.545)	**0.019**	3.950 (−9.536~17.436)	0.548
	Peak IOP	0.131 (0.668~5.593)	**0.014**	−4.172 (−17.859~9.515)	0.532
	Axial length	8.271 (−9.080~25.622)	0.338		
	CCT	−0.109 (−0.967~0.748)	0.796		
	Lens status	94.035 (9.765~178.306)	**0.030**	58.203 (−13.480~129.886)	0.106
	Number of eyedrops	16.643 (−1.934~35.219)	0.077		
	PG analogues	45.667 (−21.720~113.054)	0.177		
	VF MD	−4.557 (−6.948~−2.165)	**0.001**	−3.749 (−6.668~−0.829)	**0.014**

CCT, central corneal thickness; DBP, diastolic blood pressure; IOP, intraocular pressure; MD, mean deviation; NLR, neutrophil-to-lymphocyte ratio; PLR, platelet-to-lymphocyte ratio; SBP, systolic blood pressure; SD, standard deviation. The statistically significant variables in the regression analysis were indicated in bold.

**Table 5:** Demographics according to the presence of glaucoma in the subjects being analyzed for cytokine analysis.

Characteristics		Control (n = 30)	POAG (n = 18)	*p* Value
Age (years)		69.8 ± 9.3	66.0 ± 12.5	0.231
Sex (male/female)		9/21 (30%)	13/5 (72.2%)	**0.007**
Diabetes, n (%)		7 (23.3%)	5 (27.8%)	0.743
Hypertension, n (%)		7 (23.3%)	4 (22.2%)	1.000
Heart coronary disease, n (%)		3 (10.0%)	1 (5.6%)	1.000
Mean SBP (mmHg)		138.5 ± 13.5	143.8 ± 10.7	0.161
Mean DBP (mmHg)		77.3 ± 9.4	83.3 ± 7.2	**0.024**
SD of SBP (mmHg)		6.1 ± 4.3	7.6 ± 5.0	0.270
SD of DBP (mmHg)		4.1 ± 3.9	3.9 ± 3.8	0.882
NLR		0.6 ± 0.2	0.7 ± 0.2	0.119
PLR		112.1 ± 38.1	111.3 ± 48.8	0.953
CCT (µm)		546.6 ± 36.7	548.1 ± 40.9	0.908
Axial length (mm)		23.5 ± 0.9	24.2 ± 0.9	**0.014**
Current IOP (mmHg)		15.4 ± 2.7	27.6 ± 8.7	**<0.001**
Peak IOP (mmHg)		16.27 ± 4.1	31.1 ± 9.3	**<0.001**
SD of IOP (mmHg)		1.4 ± 1.3	4.7 ± 2.7	**<0.001**
Average RNFL thickness (µm)		79.5 ± 15.2	61.2 ± 13.8	**0.002**
Number of eyedrops		0.1 ± 0.4	3.3 ± 1.2	**<0.001**
PG analogs		2 (6.7%)	16 (88.9%)	**<0.001**
Lens status	Phakic	30 (100%)	10 (55.6%)	**<0.001**
	Pseudophakic	0 (0%)	8 (44.4%)	
Visual field 24-2	MD (dB)	−4.9 ± 3.7	−16.6 ± 9.7	**<0.001**
	PSD (dB)	2.7 ± 1.5	8.2 ± 3.4	**<0.001**

CCT, central corneal thickness; DBP, diastolic blood pressure; IOP, intraocular pressure; MD, mean deviation; NLR, neutrophil-to-lymphocyte ratio; PLR, platelet-to-lymphocyte ratio; POAG, primary open-angle glaucoma; PSD, pattern standard deviation; RNFL, retinal nerve fiber layer; SBP, systolic blood pressure; SD, standard deviation. Student’s *t*-test for continuous variables and chi-squared test for categorical variables. Statistically significant differences between groups are indicated in bold.

**Table 6:** Cytokine levels in the aqueous humor in subjects without and with glaucoma.

Cytokine (pg/mL)	Control (n = 30)	POAG (n = 18)	*p* Value
TGF-β1	62.5 ± 37.4	120.5 ± 69.3	**0.003**
TGF-β2	354.8 ± 199.1	707.5 ± 370.3	**0.001**
VEGF	96.6 ± 64.6	126.1 ± 103.1	0.254
MCP1	385.5 ± 157.9	820.1 ± 573.5	**0.005**
GM-CSF	3.4 ± 4.8	5.2 ± 0.1	0.168
CXCL1	24.4 ± 36.2	35.5 ± 42.2	0.443
INF alpha	0.9 ± 0.5	0.6 ± 0.4	0.131
IL1ra	724.5 ± 2297.9	231.0 ± 183.4	0.370
IL-6	71.2 ± 89.1	110.7 ± 196.7	0.496
IL-7	1.3 ± 1.0	3.9 ± 2.4	**<0.001**
IL-8	4.6 ± 3.9	30.3 ± 52.6	0.054
IL-15	0.7 ± 0.4	1.6 ± 0.8	**0.001**
CXCL10	35.6 ± 86.2	55.4 ± 45.1	0.376
CCL3	10.2 ± 2.4	11.5 ± 3.1	0.142
CCL4	30.8 ± 25.7	35.0 ± 24.5	0.624
PDGFAA	55.3 ± 17.9	74.5 ± 21.4	**0.002**
PD-L1	6.7 ± 2.9	15.6 ± 7.5	**<0.001**
TNFbeta	0.3 ± 0.3	0.4 ± 0.3	0.175

CCL, CC chemokine ligand; CXCL, CXC chemokine ligand; GM-CSF, granulocyte–macrophage colony-stimulating factor; IL, interleukin; MCP, macrophage chemotactic protein; PDGF, platelet-derived growth factor; PD-L1, programmed death-ligand; TGF, transforming growth factor; TNF, tumor necrosis factor; VEGF, vascular endothelial growth factor. Student’s *t*-test: Statistically significant differences between two groups were indicated in bold.

**Table 7:** The factors associated with current IOP.

Parameter	Characteristics	Univariate	*p* Value	Multivariate	*p* Value
		β Coefficient (95% CI)		β Coefficient (95% CI)	
Systemic factor	Age	−0.066 (−0.293~0.162)	0.562		
	Gender	−0.5.745 (−10.263~−1.227)	**0.014**	−0.146 (−3.969~3.677)	0.938
	Diabetes	0.583 (−4.971~6.137)	0.833		
	Hypertension	1.088 (−4.627~6.804)	0.703		
	Mean SBP (mmHg)	0.237 (0.059~0.416)	**0.010**	0.143 (−0.021~0.307)	0.086
	Mean DBP (mmHg)	0.279 (0.024~0.533)	**0.033**	−0.035 (−0.276~0.203)	0.769
	SD of SBP (mmHg)	0.131 (−0.400~0.661)	0.622		
	SD of DBP (mmHg)	−0.070 (−0.704~0.564)	0.825		
	NLR	6.829 (−3.555~17.214)	0.192		
	PLR	−0.005 (−0.053~0.063)	0.856		
Ocular factors	Axial length	1.563 (−0.553~3.679)	0.144		
	CCT	0.023 (−0.045~0.090)	0.503		
	Lens status	15.925 (11.527~20.323)	**<0.001**	12.041 (6.861~17.220)	**<0.001**
Cytokines	TGF-β1	0.070 (0.034~0.106)	**<0.001**	−0.001 (−0.038~0.035)	0.935
	TGF-β2	0.013 (0.006~0.020)	**<0.001**	0.004 (−0.003~0.011)	0.220
	MCP1	0.010 (0.006~0.015)	**<0.001**	−0.002 (−0.008~0.005)	0.599
	IL-7	2.384 (1.406~3.362)	**<0.001**	−1.194 (−2.844~0.456)	0.150
	IL-15	7.315 (4.566~10.063)	**<0.001**	−6.292 (−13.920~1.336)	0.103
	PDGFAA	0.188 (0.088~0.287)	**<0.001**	0.016 (−0.106~0.139)	0.790
	PD-L1	0.891 (0.638~1.144)	**<0.001**	1.363 (0.627~2.099)	0.001

CCT, central corneal thickness; DBP, diastolic blood pressure; IOP, intraocular pressure; IL, interleukin; MCP, macrophage chemotactic protein; NLR, neutrophil-to-lymphocyte ratio; PDGF, platelet-derived growth factor; PD-L1, programmed death-ligand; PLR, platelet-to-lymphocyte ratio; SBP, systolic blood pressure; SD, standard deviation; TGF, transforming growth factor. The statistically significant variables in the regression analysis were indicated in bold.

## Data Availability

The data presented in this study are available on request from the corresponding author due to privacy.

## Supplementary Materials

The following supporting information can be downloaded at: https://www.mdpi.com/article/10.3390/cells13121030/s1, Figure S1: Principal Component Analysis (PCA) of Exosome Profiles in primary open angle glaucoma (POAG) and Control Groups; Figure S2: Principal Component Analysis (PCA) of Cytokine Profiles in primary open angle glaucoma (POAG) and Control Groups; Table S1: Univariate and multivariate analysis of factors associated with IOP; Table S2: Univariate and multivariate analysis of factors associated with SD of IOP; Table S3: The factors associated with SD of IOP; Table S4: The factors associated with PDL-1; Table S5: The factors associated with mean deviation of visual field.
