# Supplementary material for: Parallel Analysis of Exosomes and Cytokines in Aqueous Humor Samples to Evaluate Biomarkers for Glaucoma
Source: Cells. 2024 Jun 13;13(12):1030. doi: 10.3390/cells13121030 (PMC11202053; doi:10.3390/cells13121030)
Supplement: Supplementary file 1 [file cells-13-01030-s001.zip › cells-2970137-supplementary.pdf]

**Supplemental Table S1**. Univariate and multivariate analysis of factors associated with IOP

| | | Univariate | | Multivariate | |
|---|---|---|---|---|---|
| Parameter | | β coefficient (95% CI) | *P* value | β coefficient (95% CI) | *P* value |
| Systemic factors | Age | -0.375(-0.738~-0.014) | **0.042** | -0.375(-0.715~-0.034) | **0.032** |
| | Gender | 0.500(-8.611~9.611) | 0.912 | | |
| | Diabetes | 1.928(-8.792~12.647) | 0.717 | | |
| | Hypertension | -0.411(-12.362~11.541) | 0.945 | | |
| | Heart Coronary | -8.591(-35.38118.200) | 0.518 | | |
| | Mean SBP (mmHg) | -0.054(-0.389~0.281) | 0.745 | | |
| | Mean DBP (mmHg) | -0.082(-0.719~0.556) | 0.796 | | |
| | SD of SBP (mmHg) | -0.281(-1.099~0.537) | 0.490 | | |
| | SD of DBP (mmHg) | -0.579(-1.688~0.531) | 0.296 | | |
| | NLR | 1.062(-1.959~4.083) | 0.479 | | |
| | PLR | 0.015(-0.061~0.092) | 0.688 | | |
| Ocular factors | Axial length | 2.501(0.073~4.929) | **0.044** | 1.100(-1.238~3.439) | 0.343 |
| | CCT | -0.052(-0.204~0.100) | 0.490 | | |
| | Lens status | 12.744(1.708~23.781) | **0.025** | 10.293(-2.623~23.209) | 0.114 |
| Exosome particle counts | CD 63 (total) | 0.034(0.003~0.065) | **0.034** | | |
| | CD 63 (CD63) | 0.053(0.009~0.096) | **0.019** | 0.042(-0.004~0.089) | 0.074 |
| | CD 81 (total) | 0.030(-0.017~0.077) | 0.202 | | |
| Exosome diameter | CD 63 | -0.532(-1.347~0.282) | 0.193 | | |
| | CD 81 | -0.751(-1.467~-0.035) | **0.040** | | |
| | CD 9 | -0.660(-1.632~0.313) | 0.177 | | |
| Exosome volume | CD 63 | 0.014(-0.002~0.030) | 0.079 | | |
| | CD 81 | 0.010(-0.015~0.036) | 0.423 | | |
| | CD 9 | 0.008(-0.008~0.024) | 0.312 | | |

CCT, Central corneal thickness; DBP, diastolic blood pressure; IOP, intraocular pressure; MD, mean deviation; NLR, neutrophil-to lymphocyte ratio; PLR, platelet-to lymphocyte ratio; SBP, systolic blood pressure; SD, standard deviation

**Supplemental Table S2.** Univariate and multivariate analysis of factors associated with SD of IOP

| | | Univariate | | Multivariate | |
|---|---|---|---|---|---|
| Parameter | | β coefficient (95% CI) | *P* value | β coefficient (95% CI) | *P* value |
| Systemic factors | Age | -0.159(-0.317~-0.002) | **0.048** | -0.097(-0.255~-0.061) | 0.205 |
| | Gender | 1.305(-3.426~~6.036) | 0.568 | | |
| | Diabetes | 0.812(-4.529~6.152) | 0.752 | | |
| | Hypertension | -2.516(-8.157~3.125) | 0.360 | | |
| | Heart Coronary | -4.003(-14.366~6.360) | 0.426 | | |
| | Mean SBP (mmHg) | -0.101(-0.261~0.060) | 0.203 | | |
| | Mean DBP (mmHg) | -0.203(-0.522~0.116) | 0.197 | | |
| | SD of SBP (mmHg) | 0.049(-0.367~0.465) | 0.806 | | |
| | SD of DBP (mmHg) | -0.349(-0.934~0.237) | 0.226 | | |
| | NLR | 0.706(-0.621~2.032) | 0.277 | | |
| | PLR | 0.025(-0.021~0.071) | 0.263 | | |
| Ocular factors | Axial length | 1.442(0.156~2.727) | **0.030** | 0.629(-0.636~1.893) | 0.297 |
| | CCT | 0.008(-0.091~0.107) | 0.866 | | |
| | Lens status | 1.619(-3.674~6.911) | 0.527 | -0.665(-5.941~4.612) | 0.787 |
| Exosome particle counts | CD 63 (total) | 0.014(0.001~0.026) | **0.034** | | |
| | CD 63 (CD63) | 0.023(0.006~0.040) | **0.011** | 0.018(-0.002~0.039) | 0.077 |
| | CD 81 (total) | 0.012(-0.007~0.031) | 0.187 | | |
| Exosome diameter | CD 63 | -0.253(-0.589~0.083) | 0.130 | | |
| | CD 81 | -0.439(-0.750~-0.128) | **0.008** | -0.203(-0.569~0164) | 0.249 |
| | CD 9 | -0.313(-0.777~0.150) | 0.172 | | |
| Exosome volume | CD 63 | 0.006(-0.001~0.012) | 0.074 | | |
| | CD 81 | 0.004(-0.007~0.014) | 0.476 | | |
| | CD 9 | 0.005(-0.001~0.011) | 0.075 | | |

CCT, Central corneal thickness; DBP, diastolic blood pressure; IOP, intraocular pressure; MD, mean deviation; NLR, neutrophil-to lymphocyte ratio; PLR, platelet-to lymphocyte ratio; SBP, systolic blood pressure; SD, standard deviation

**Supplemental Table S3.** The factors associated with SD of IOP

| | | Univariate | | Multivariate | |
|---|---|---|---|---|---|
| Parameter | | β coefficient (95% CI) | *P* value | β coefficient (95% CI) | *P* value |
| Systemic factor | Age | -0.065(-0.142~0.012) | 0.096 | | |
| | Gender | -2.149(-3.698~-0.599) | **0.008** | | 0.483 |
| | Diabetes | 0.870(-1.350~3.089) | 0.433 | | |
| | Hypertension | -0.172(-2.297~1.953) | 0.870 | | |
| | Mean SBP (mmHg) | 0.043(-0.030~0.116) | 0.236 | | |
| | Mean DBP (mmHg) | -0.004(-0.104~0.095) | 0.927 | | |
| | SD of SBP (mmHg) | -0.007(-0.186~0.171) | 0.933 | | |
| | SD of DBP (mmHg) | -0.040(-0.257~0.177) | 0.711 | | |
| | NLR | 1.079(-2.455~4.613) | 0.540 | | |
| | PLR | -0.009(-0.028~0.010) | 0.337 | | |
| Ocular factors | Axial length | 0.568(-0.302~1.438) | 0.193 | | |
| | CCT | 0.003(-0.021~0.026) | 0.807 | | |
| | Lens status | 3.386(1.575~5.198) | **0.001** | 1.647(-0.694~3.988) | 0.160 |
| Cytokines | TGF-β1 | 0.018(0.005~0.031) | **0.008** | -0.008(-0.025~0.009) | 0.355 |
| | TGF-β2 | 0.004(0.002~0.006) | **0.001** | 0.002(-0.001~0.006) | 0.173 |
| | MCP1 | 0.004(0.002~0.005) | **<0.001** | 0.002(-0.001~0.004) | 0.267 |
| | IL-7 | 0.696(0.382~1.010) | **<0.001** | -0.025(-0.756~0.705) | 0.944 |
| | IL-15 | 2.304(1.403~3.205) | **<0.001** | -2.258(-6.099~1.583) | 0.237 |
| | PDGFAA | 0.063(0.031~0.096) | **<0.001** | 0.008(-0.045~0.060) | 0.762 |
| | PD-L1 | 0.263(0.180~0.347) | **<0.001** | 0.354(0.002~0.707) | **0.049** |

CCT, Central corneal thickness; DBP, diastolic blood pressure; IOP, intraocular pressure; IL, interleukin; MCP, macrophage chemotactic protein; MD, mean deviation; NLR, neutrophil-to lymphocyte ratio; PDGF, platelet-derived growth factor; PD-L1, programmed death-ligand; PLR, platelet-to lymphocyte ratio; SBP, systolic blood pressure; SD, standard deviation; TGF, transforming growth factor

**Supplemental Table S4.** The factors associated with PDL-1

| Parameter | | Univariate | | Multivariate | |
|---|---|---|---|---|---|
| | | β coefficient (95% CI) | *P* value | β coefficient (95% CI) | *P* value |
| Systemic factors | Age | -0.020(-0.205~0.166) | 0.833 | | |
| | Gender | -5.513(-9.070~-1.955) | **0.003** | -0.980(-4.295~2.334) | 0.551 |
| | Diabetes | 1.244(-3.247~5.734) | 0.580 | | |
| | Hypertension | 1.523(-3.097~6.143) | 0.510 | | |
| | Mean SBP (mmHg) | 0.056(-0.099~0.210) | 0.471 | | |
| | Mean DBP (mmHg) | 0.171(-0.040~0.382) | 0.110 | | |
| | SD of SBP (mmHg) | 0.082(-0.348~0.513) | 0.702 | | |
| | SD of DBP (mmHg) | -0.030(-0.544~0.484) | 0.907 | | |
| | NLR | 3.099(-5.431~11.6269) | 0.468 | | |
| | PLR | -0.019(-0.066~0.028) | 0.418 | | |
| Ocular factors | Current IOP | 0.586(0.420~0.752) | **<0.001** | 0.180(-0.100~0.460) | 0.200 |
| | Peak IOP | 0.525(0.389~0.661) | **<0.001** | | |
| | SD of IOP | 1.965(1.342~2.588) | **<0.001** | 0.899(0.164~1.634) | **0.018** |
| | Axial length | 2.728(0.904~4.552) | 0.004 | | |
| | CCT | 0.008(-0.049~0.065) | 0.789 | | |
| | Lens status | 10.698(6.536~14.861) | **<0.001** | 1.693(0.497~-3.3322) | 0.497 |
| | Number of eyedrops | 2.636(1.805~3.467) | **<0.001** | 1.135(-0.666~2.935) | 0.209 |
| | PG analogues | 8.463(5.311~11.614) | **<0.001** | 0.526(-5.235~6.287) | 0.854 |

CCT, Central corneal thickness; DBP, diastolic blood pressure; IOP, intraocular pressure; LMR, lymphocyte-to monocyte ratio; MD, mean deviation; NLR, neutrophil-to lymphocyte ratio; PG, prostaglandin; PLR, platelet-to lymphocyte ratio; PSD, pattern standard deviation; SBP, systolic blood pressure; SD, standard deviation

**Supplemental Table S5.** The factors associated with mean deviation of visual field

| | | Univariate | | Multivariate | |
|---|---|---|---|---|---|
| Parameter | | β coefficient (95% CI) | *P* value | β coefficient (95% CI) | *P* value |
| Systemic factors | Age | -0.142(-0.455~0.171) | 0.362 | | |
| | Gender | 7.635(0.949~14.320) | **0.027** | 3.390 (-5.728~8.725) | 0.665 |
| | Diabetes | 0.374(-8.225~8.973) | 0.930 | | |
| | Hypertension | -0.707(-9.303~7.889) | 0.868 | | |
| | Mean SBP (mmHg) | -0.189(-0.456~0.0749) | 0.160 | | |
| | Mean DBP (mmHg) | -0.284(-0.704~0.136) | 0.177 | | |
| | SD of SBP (mmHg) | 0.205(-0.562~0.972) | 0.589 | | |
| | SD of DBP (mmHg) | 0.250(-0.800~1.301) | 0.630 | | |
| | NLR | -3.305(-20.067~13.457) | 0.690 | | |
| | PLR | -0.019(-0.100~0.063) | 0.642 | | |
| Ocular factors | Current IOP | -0.738(-1.008~-0.467) | **<0.001** | -0.155(-0.834~0.525) | 0.635 |
| | Peak IOP | -0.548(-0.817~-0.279) | **<0.001** | | |
| | SD of IOP | -1.720(-2.981~-0.459) | **0.009** | 0.057(-1.764~1.879) | 0.947 |
| | Axial length | -3.032(-6.411~0.347) | 0.077 | | |
| | CCT | 0.048(-0.047~0.142) | 0.306 | | |
| | Lens status | -15.980(-21.521~-10.438) | **<0.001** | -7.013(-17.907~3.881) | 0.190 |
| | Number of eyedrops | -3.134(-4.808~-1.459) | **0.001** | -0.031(-3.399~3.338) | 0.985 |
| | PG analogues | -9.348(-15.712~-2.984) | **0.005** | -0.901(-11.588~9.786) | 0.860 |
| Cytokines | TGF-β1 | -0.083(-0.132~-0.034) | **0.002** | -0.028(-0.097~0.040) | 0.394 |
| | TGF-β2 | -0.009(-0.019~0.002) | 0.097 | | |
| | MCP1 | -0.009(-0.016~-0.002) | **0.012** | 0.012(-0.003~0.027) | 0.105 |
| | IL-7 | -2.532(-3.808~-1.256) | **<0.001** | -1.207(-4.290~1.876) | 0.417 |
| | IL-15 | -9.304(-12.163~-4.445) | **<0.001** | -0.096(-16.877~16.685) | 0.990 |
| | PDGFAA | -0.229(-0.394~-0.063) | **0.008** | -0.116(-0.346~0.113) | 0.297 |
| | PD-L1 | -0.853(-1.247~-0.458) | **<0.001** | -0.382(-2.178~1.414) | 0.657 |

CCT, Central corneal thickness; DBP, diastolic blood pressure; IOP, intraocular pressure; IL, interleukin; MCP, macrophage chemotactic protein; MD, mean deviation; NLR, neutrophil-to lymphocyte ratio; PDGF, platelet-derived growth factor; PD-L1, programmed death-ligand; PLR, platelet-to lymphocyte ratio; SBP, systolic blood pressure; SD, standard deviation; TGF, transforming growth factor

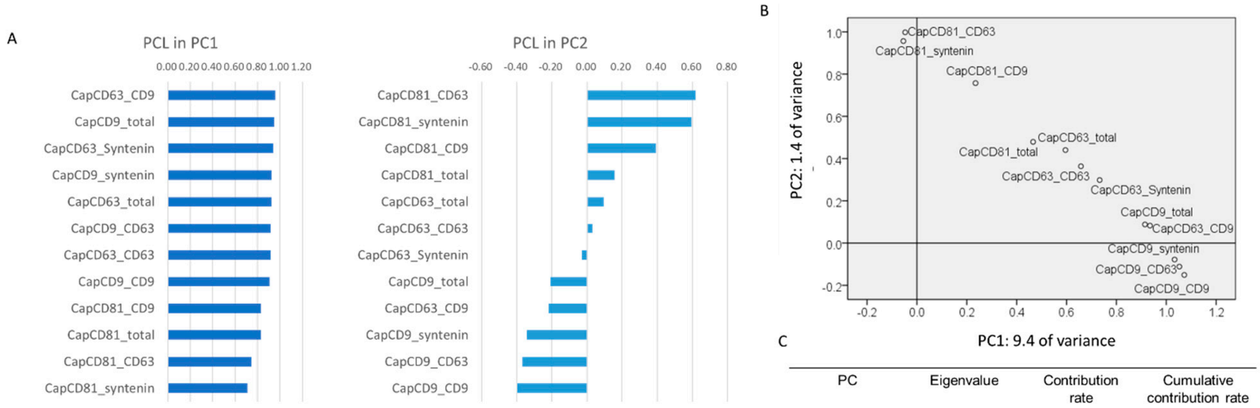


C

| PC | Eigenvalue | Contribution rate | Cumulative contribution rate |
|---|---|---|---|
| 1 | 9.359 | 78.0% | 78.0% |
| 2 | 1.428 | 11.9% | 89.9% |

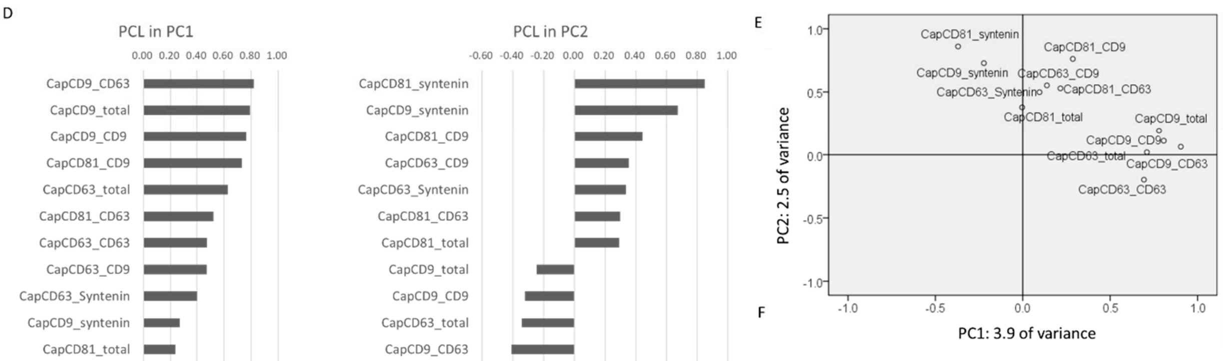


F

| PC | Eigenvalue | Contribution rate | Cumulative contribution rate |
|---|---|---|---|
| 1 | 3.877 | 32.3% | 32.3% |
| 2 | 2.489 | 20.7% | 53.0% |

**Supplemental Figure S1**. Principal Component Analysis (PCA) of Exosome Profiles in primary open angle glaucoma (POAG) and Control Groups

(A)&(D): Principal component loadings (PCL) for the first (PC1) and second (PC2) principal components in POAG patients (A) and control subjects (D). These panels illustrate the weights of variables contributing to each principal component. (B)&(E): Biplots of PCL in PC1 and PC2 for POAG patients (B) and controls (E). (C)&(F): Eigenvalues, contribution rates (CR), and cumulative contribution rates (CCR) for PC1 and PC2 in the POAG group (C) and control group (F).

Cumulative contribution rate, CCR; contribution rates, CR; PC, principal component

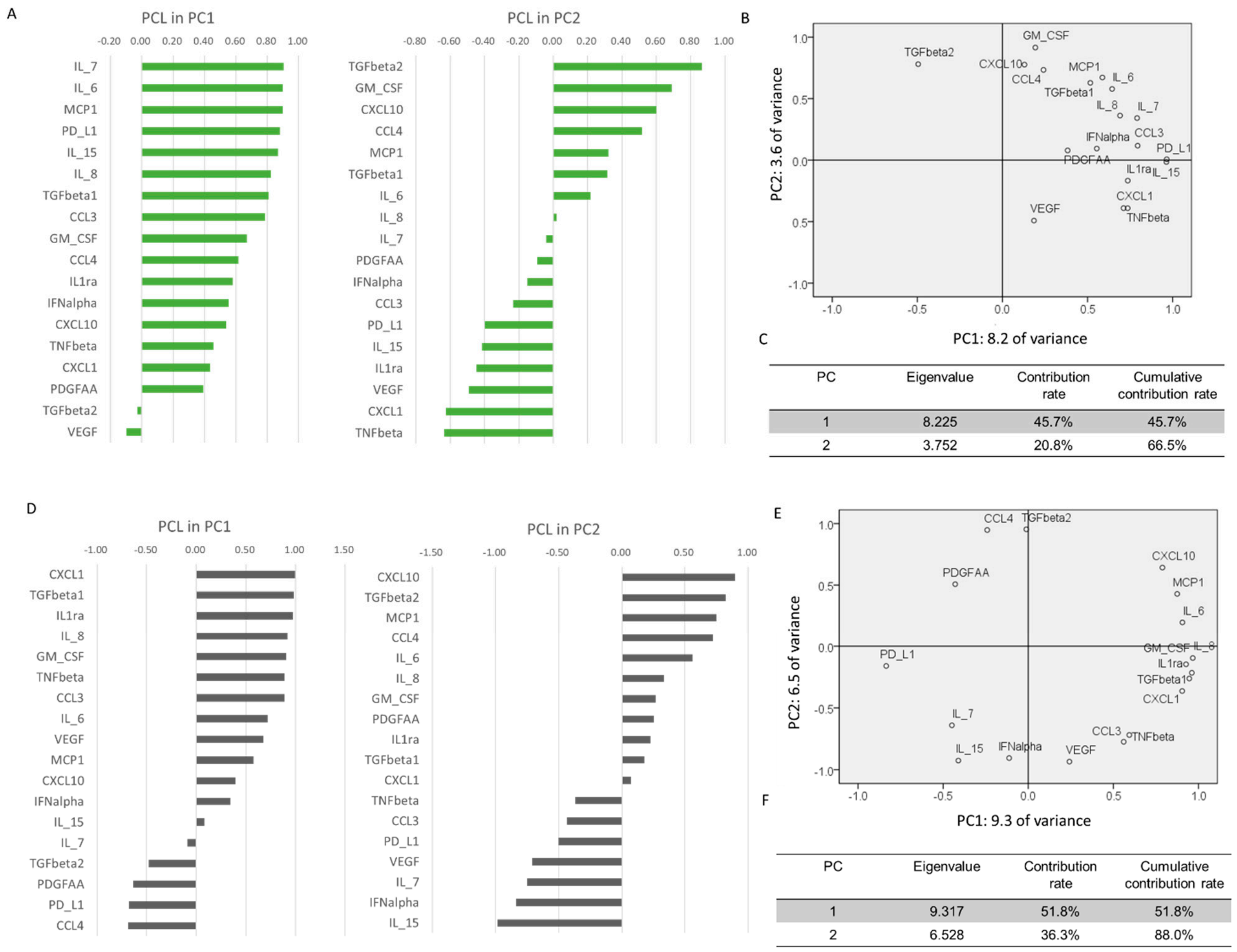


C

| PC | Eigenvalue | Contribution rate | Cumulative contribution rate |
|---|---|---|---|
| 1 | 8.225 | 45.7% | 45.7% |
| 2 | 3.752 | 20.8% | 66.5% |

F

| PC | Eigenvalue | Contribution rate | Cumulative contribution rate |
|---|---|---|---|
| 1 | 9.317 | 51.8% | 51.8% |
| 2 | 6.528 | 36.3% | 88.0% |

**Supplemental Figure S2**. Principal Component Analysis (PCA) of Cytokine Profiles in primary open angle glaucoma (POAG) and Control Groups

(A)&(D): Principal component loadings (PCL) for the first (PC1) and second (PC2) principal components in POAG patients (A) and control subjects (D). These panels illustrate the weights of variables contributing to each principal component. (B)&(E): Biplots of PCL in PC1 and PC2 for POAG patients (B) and controls (E). (C)&(F): Eigenvalues, contribution rates (CR), and cumulative contribution rates (CCR) for PC1 and PC2 in the POAG group (C) and control group (F).

Cumulative contribution rate, CCR; contribution rates, CR; PC, principal component
